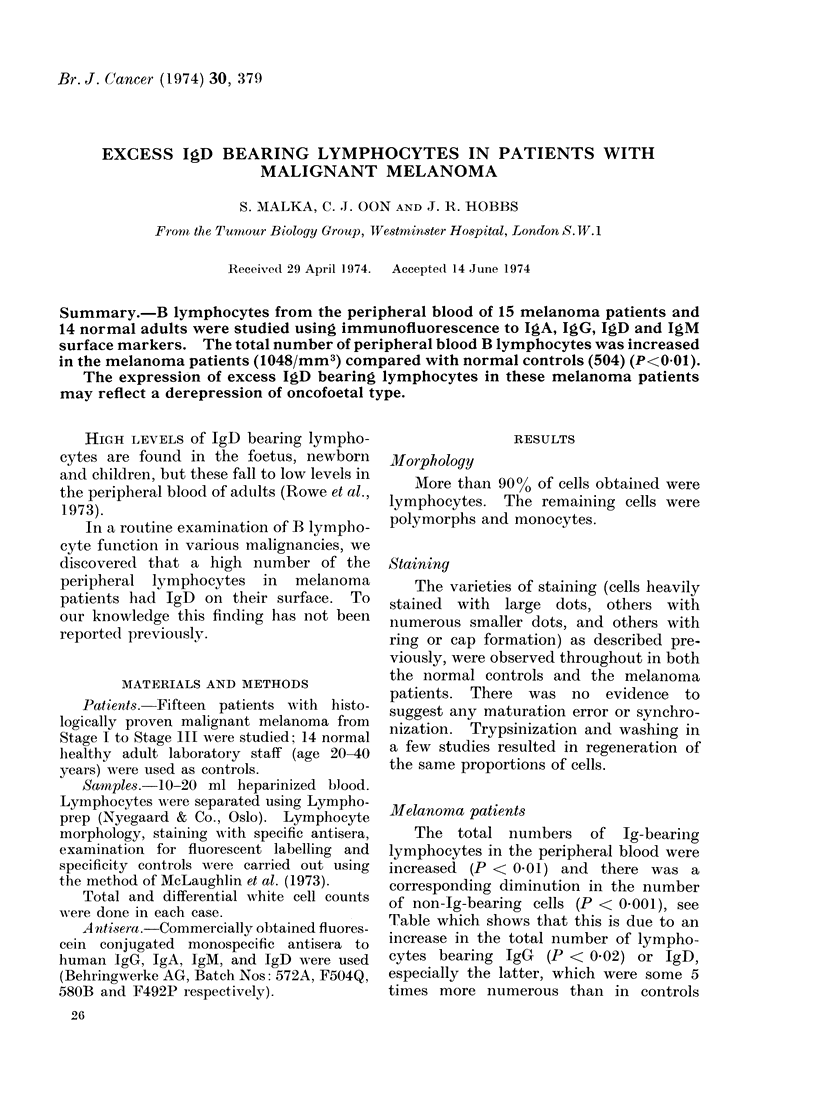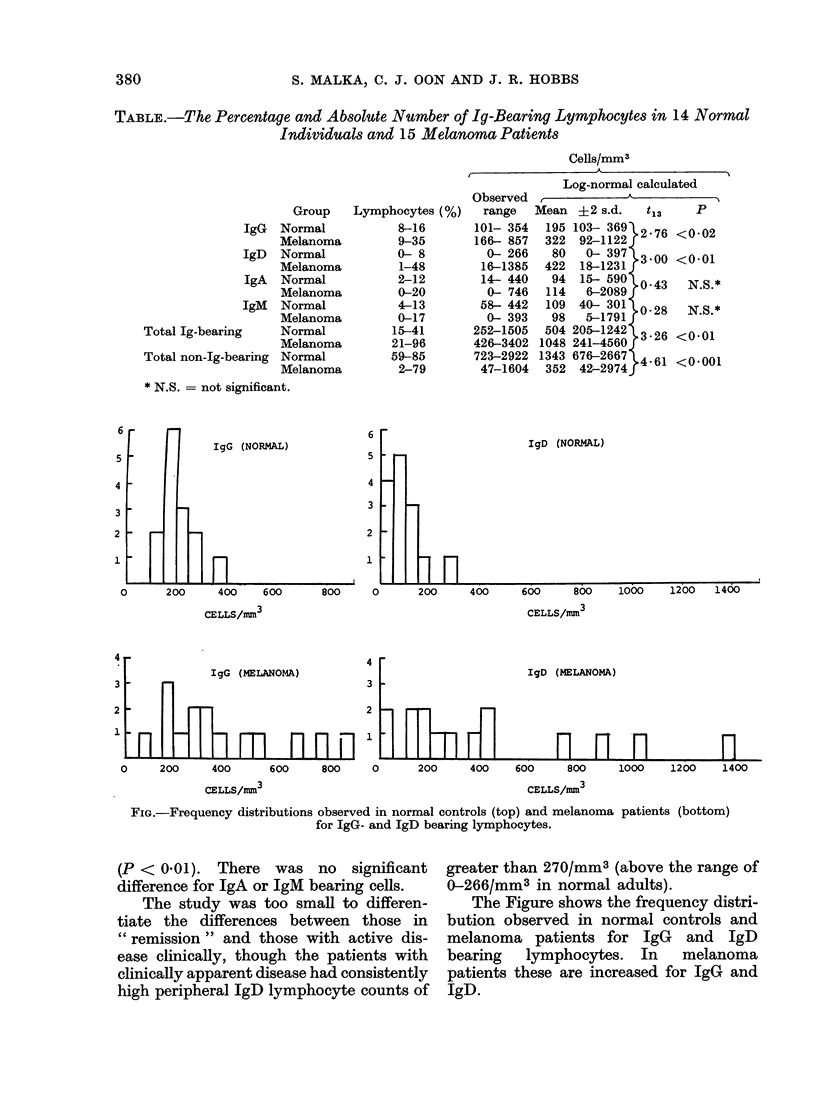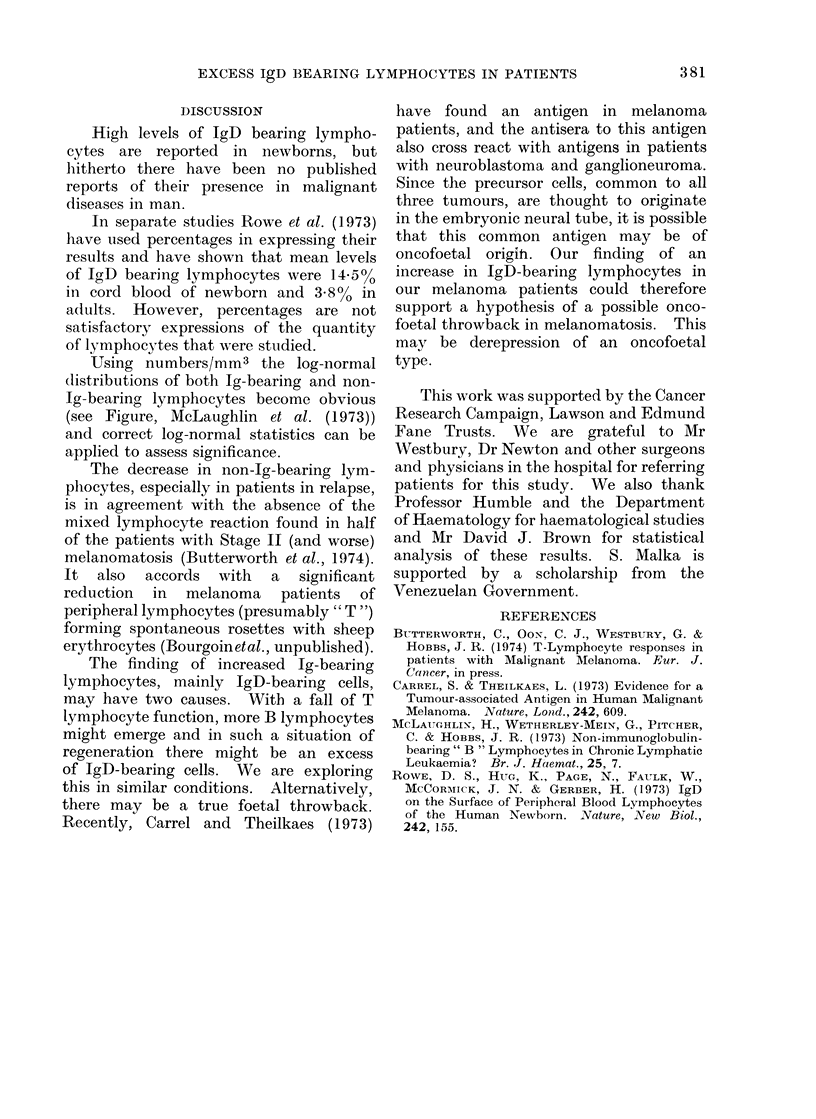# Excess IgD Bearing Lymphocytes in Patients with Malignant Melanoma

**DOI:** 10.1038/bjc.1974.211

**Published:** 1974-11

**Authors:** S. Malka, C. J. Oon, J. R. Hobbs

## Abstract

B lymphocytes from the peripheral blood of 15 melanoma patients and 14 normal adults were studied using immunofluorescence to IgA, IgG, IgD and IgM surface markers. The total number of peripheral blood B lymphocytes was increased in the melanoma patients (1048/mm^3^) compared with normal controls (504) (*P*<0·01).

The expression of excess IgD bearing lymphocytes in these melanoma patients may reflect a derepression of oncofoetal type.


*Br. J. Cancer* (1974) **30**, 379

### EXCESS IgD BEARING LYMPHOCYTES IN PATIENTS WITH MALIGNANT MELANOMA

S. MALKA, C. J. OON AND J. R. HOBBS

*From the Tumour Biology Group, Westminster Hospital, London S.W.1*

Received 29 April 1974. Accepted 14 June 1974

**Summary.—B lymphocytes from the peripheral blood of 15 melanoma patients and 14 normal adults were studied using immunofluorescence to IgA, IgG, IgD and IgM surface markers. The total number of peripheral blood B lymphocytes was increased in the melanoma patients (1048/mm$^3$) compared with normal controls (504) ($P<0\cdot01$).**

**The expression of excess IgD bearing lymphocytes in these melanoma patients may reflect a derepression of oncofoetal type.**

HIGH LEVELS of IgD bearing lymphocytes are found in the foetus, newborn and children, but these fall to low levels in the peripheral blood of adults (Rowe *et al.*, 1973).

In a routine examination of B lymphocyte function in various malignancies, we discovered that a high number of the peripheral lymphocytes in melanoma patients had IgD on their surface. To our knowledge this finding has not been reported previously.

### MATERIALS AND METHODS

*Patients.*—Fifteen patients with histologically proven malignant melanoma from Stage I to Stage III were studied; 14 normal healthy adult laboratory staff (age 20–40 years) were used as controls.

*Samples.*—10–20 ml heparinized blood. Lymphocytes were separated using Lymphoprep (Nyegaard & Co., Oslo). Lymphocyte morphology, staining with specific antisera, examination for fluorescent labelling and specificity controls were carried out using the method of McLaughlin *et al.* (1973).

Total and differential white cell counts were done in each case.

*Antisera.*—Commercially obtained fluorescein conjugated monospecific antisera to human IgG, IgA, IgM, and IgD were used (Behringwerke AG, Batch Nos: 572A, F504Q, 580B and F492P respectively).

### RESULTS

### *Morphology*

More than 90% of cells obtained were lymphocytes. The remaining cells were polymorphs and monocytes.

### *Staining*

The varieties of staining (cells heavily stained with large dots, others with numerous smaller dots, and others with ring or cap formation) as described previously, were observed throughout in both the normal controls and the melanoma patients. There was no evidence to suggest any maturation error or synchronization. Trypsinization and washing in a few studies resulted in regeneration of the same proportions of cells.

### *Melanoma patients*

The total numbers of Ig-bearing lymphocytes in the peripheral blood were increased ($P < 0\cdot01$) and there was a corresponding diminution in the number of non-Ig-bearing cells ($P < 0\cdot001$), see Table which shows that this is due to an increase in the total number of lymphocytes bearing IgG ($P < 0\cdot02$) or IgD, especially the latter, which were some 5 times more numerous than in controls

TABLE.—*The Percentage and Absolute Number of Ig-Bearing Lymphocytes in* 14 *Normal Individuals and* 15 *Melanoma Patients*

| | | | Cells/mm³ | | | | |
|---|---|---|---|---|---|---|---|
| | | | Observed | Log-normal calculated | | | |
| | Group | Lymphocytes (%) | range | Mean | ±2 s.d. | $t_{13}$ | $P$ |
| IgG | Normal | 8–16 | 101– 354 | 195 | 103– 369 | 2·76 | <0·02 |
| | Melanoma | 9–35 | 166– 857 | 322 | 92–1122 | | |
| IgD | Normal | 0– 8 | 0– 266 | 80 | 0– 397 | 3·00 | <0·01 |
| | Melanoma | 1–48 | 16–1385 | 422 | 18–1231 | | |
| IgA | Normal | 2–12 | 14– 440 | 94 | 15– 590 | 0·43 | N.S.* |
| | Melanoma | 0–20 | 0– 746 | 114 | 6–2089 | | |
| IgM | Normal | 4–13 | 58– 442 | 109 | 40– 301 | 0·28 | N.S.* |
| | Melanoma | 0–17 | 0– 393 | 98 | 5–1791 | | |
| Total Ig-bearing | Normal | 15–41 | 252–1505 | 504 | 205–1242 | 3·26 | <0·01 |
| | Melanoma | 21–96 | 426–3402 | 1048 | 241–4560 | | |
| Total non-Ig-bearing | Normal | 59–85 | 723–2922 | 1343 | 676–2667 | 4·61 | <0·001 |
| | Melanoma | 2–79 | 47–1604 | 352 | 42–2974 | | |

* N.S. = not significant.

FIG.—Frequency distributions observed in normal controls (top) and melanoma patients (bottom) for IgG- and IgD bearing lymphocytes.

($P < 0{\cdot}01$). There was no significant difference for IgA or IgM bearing cells.

The study was too small to differentiate the differences between those in " remission " and those with active disease clinically, though the patients with clinically apparent disease had consistently high peripheral IgD lymphocyte counts of greater than 270/mm³ (above the range of 0–266/mm³ in normal adults).

The Figure shows the frequency distribution observed in normal controls and melanoma patients for IgG and IgD bearing lymphocytes. In melanoma patients these are increased for IgG and IgD.

### DISCUSSION

High levels of IgD bearing lymphocytes are reported in newborns, but hitherto there have been no published reports of their presence in malignant diseases in man.

In separate studies Rowe *et al.* (1973) have used percentages in expressing their results and have shown that mean levels of IgD bearing lymphocytes were 14·5% in cord blood of newborn and 3·8% in adults. However, percentages are not satisfactory expressions of the quantity of lymphocytes that were studied.

Using numbers/$mm^3$ the log-normal distributions of both Ig-bearing and non-Ig-bearing lymphocytes become obvious (see Figure, McLaughlin *et al.* (1973)) and correct log-normal statistics can be applied to assess significance.

The decrease in non-Ig-bearing lymphocytes, especially in patients in relapse, is in agreement with the absence of the mixed lymphocyte reaction found in half of the patients with Stage II (and worse) melanomatosis (Butterworth *et al.*, 1974). It also accords with a significant reduction in melanoma patients of peripheral lymphocytes (presumably " T ") forming spontaneous rosettes with sheep erythrocytes (Bourgoin *et al.*, unpublished).

The finding of increased Ig-bearing lymphocytes, mainly IgD-bearing cells, may have two causes. With a fall of T lymphocyte function, more B lymphocytes might emerge and in such a situation of regeneration there might be an excess of IgD-bearing cells. We are exploring this in similar conditions. Alternatively, there may be a true foetal throwback. Recently, Carrel and Theilkaes (1973) have found an antigen in melanoma patients, and the antisera to this antigen also cross react with antigens in patients with neuroblastoma and ganglioneuroma. Since the precursor cells, common to all three tumours, are thought to originate in the embryonic neural tube, it is possible that this common antigen may be of oncofoetal origin. Our finding of an increase in IgD-bearing lymphocytes in our melanoma patients could therefore support a hypothesis of a possible oncofoetal throwback in melanomatosis. This may be derepression of an oncofoetal type.

This work was supported by the Cancer Research Campaign, Lawson and Edmund Fane Trusts. We are grateful to Mr Westbury, Dr Newton and other surgeons and physicians in the hospital for referring patients for this study. We also thank Professor Humble and the Department of Haematology for haematological studies and Mr David J. Brown for statistical analysis of these results. S. Malka is supported by a scholarship from the Venezuelan Government.

### REFERENCES

BUTTERWORTH, C., OON, C. J., WESTBURY, G. & HOBBS, J. R. (1974) T-Lymphocyte responses in patients with Malignant Melanoma. *Eur. J. Cancer*, in press.

CARREL, S. & THEILKAES, L. (1973) Evidence for a Tumour-associated Antigen in Human Malignant Melanoma. *Nature, Lond.*, **242**, 609.

MCLAUGHLIN, H., WETHERLEY-MEIN, G., PITCHER, C. & HOBBS, J. R. (1973) Non-immunoglobulin-bearing " B " Lymphocytes in Chronic Lymphatic Leukaemia? *Br. J. Haemat.*, **25**, 7.

ROWE, D. S., HUG, K., PAGE, N., FAULK, W., MCCORMICK, J. N. & GERBER, H. (1973) IgD on the Surface of Peripheral Blood Lymphocytes of the Human Newborn. *Nature, New Biol.*, **242**, 155.